# Supplementary material for: Modeling SARS-CoV-2 antibody seroprevalence and its determinants in Ghana: A nationally representative cross-sectional survey
Source: PLOS Glob Public Health. 2023 May 5;3(5):e0001851. doi: 10.1371/journal.pgph.0001851 (PMC10162519; doi:10.1371/journal.pgph.0001851)
Supplement: Attachment — Submitted filename: Response to Reviewers.doc [file pgph.0001851.s001.doc]

**NOGUCHI MEMORIAL INSTITUTE**

**FOR MEDICAL RESEARCH (NMIMR)**

**COLLEGE OF HEALTH SCIENCES**

DEPARTMENT OF EPIDEMIOLOGY

14^th^ February 2023

The Editor

PLOS Global Public Health

Dear Editor,

**Responses To Points Raised by Editor and Reviewers**

In response to the points raised by the editor and reviewers please see below our responses

**Journal Requirements**

1. Please amend your Financial Disclosure statement. This is published with the article. It must therefore be completed in full sentences and contain the exact wording you wish to be published

**Response**: The financial disclosure statement has been amended

a. Please clarify all sources of funding (financial or material) for your study. List the grants (with grant number) or organizations (with URL) that supported your study, including funding received from your institution

**Response**: The sources of funding for the study have been listed under “Role of Funding Source”.

b. State the initials alongside each funding source, of each author to receive the grant

**Response**: This has been done.

c. State what role the funders took in the study. If the funders had no role in your study please state, “The funders had no role in study design, data collection and analysis, decision to publish, or preparation of the manuscript”.

**Response**: This has been done.

d. If any authors received a salary from any of your funders, please state which authors an which funders

**Response**: None of the authors received salary from the funders

2. Please provide separate figure files in tiff or eps format

**Respons**e: Figure file has been provided in tiff format

3. We do not publish any copyright or trademark symbols that usually accompany proprietary names, eg (R), (C), or TM (eg next to drug or reagent names). Please remove all instances of trademark/copyright symbols throughout the text, including R on page 13.

**Response**: All instances of trademark/copyright symbols have been removed from the text

4. Some material included in your submission may be copyrighted. According to PLOS’s copyright policy, authors who use figures or other material (e.g., graphics, clipart, maps) from another author or copyright holder must demonstrate or obtain permission to publish this material under the Creative Commons Attribution 4.0 International (CC BY 4.0) License used by PLOS journals. Please closely review the details of PLOS’s copyright requirements here: PLOS Licenses and Copyright. If you need to request permissions from a copyright holder, you may use PLOS's Copyright Content Permission form.Please respond directly to this email or email the journal office and provide any known details concerning your material's license terms and permissions required for reuse, even if you have not yet obtained copyright permissions or are unsure of your material's copyright compatibility.

**Response**: The copyright response has been sent separately by email.

**Reviewer #1**

1. When was the survey done? Methods section mentions it as between February and December 2021 and is approximately 11 months. COVID-19 seroprevalence estimates needs to be generated within a shorter period of time to have policy implications. What inferences can be made out from seroprevalence estimates calculated over a 11-month period and how useful will be the estimates?

**Response:** The study was done over an 11-month period but samples collection was done at three timepoints, February-March, May-July and November-December. Seroprevalence estimates over an 11-month period shows that people were continually being exposed to the virus and transmission was ongoing in the country.

2. Figure 1. Colors indicating the transmission zones are not mentioned and hence could not make out the difference. If black dot represents the clusters where the survey was carried out, it shows that the survey was not representative. More clusters in the south than the north. The second regional boundary from bottom, has more clusters surveyed than the rest.

**Response:** Colors indicating the transmission zones have been indicated in the legend. Transmission across Ghana was not equal and the selection of areas to be included in the study was based on data from the Ghana Health Service (GHS) which was all based on the prevalence of infection. There was no data on seroprevalence in Ghana as at the time of conception and commencement of the study thus the reliance on data from the GHS.

3. What was a cluster? Was EA considered a cluster? Kindly clarify

**Response:** Yes, an EA was considered as a cluster in this study since each EA contained a list of households from which we sampled

4. How many members from each household were selected? was it one or more than one? Mention it explicitly.

**Response:** From each household, one person was selected to be included in the study. This has been written out in the text.

5. Authors assumed 5% seroprevalence among general population for sample size calculation. How come they arrived at a sample size for households instead of individuals? Kindly explain

**Response**: Yes, we arrived at the sample size of 6000 households because we only selected one person from each household which in this case means that if you have 6000 people based on the sample size formula, then you required a total of 6000 households to reach out to the number of people. That is, the sample size for the number of individuals is the same as the number of households selected

6. Also, how were the 6000 households distributed among the transmission zones. From the results, it is evident that the distribution was not uniform. Kindly detail the sampling procedure followed for the survey. Current explanation lacks clarity.

**Response:** This study used sampling probability proportionate to size of the strata. The size of the strata were not the same based on transmission of the virus within the country.

7. Why were samples of nasopharyngeal swabs taken for a seroprevalence study? Please explain

**Response:** Nasopharyngeal samples were collected to determine infection. The results are presented in another manuscript that’s focused on infection and circulating variants

8. Urban population constitutes 50-55% in Ghana. However, 71% of the surveyed population were from Urban estimates. Since, COVID-19 pandemic initially spread in cities, surveying a greater number of people in Urban areas might overestimate the seroprevalence. How did the authors address this concern?

**Response**: The statistical analysis adjusted for sampling weight to address the issues of oversampling

9. When a sample size is calculated for a strata, we expect sample sizes equal across the strata? Why were the number of individuals enrolled for the study were different across strata?

**Response**: This was done because the size of strata varied according to the data obtained from the Ghana Health Service where the country was zoned into 4 – high burden, medium burden, low burden and very low burden areas.

10. Vaccinated individuals were not included in the analysis? Can authors explain why was this done? If they had to be dropped from analysis, why were they included in the survey? Kindly state the objective of the survey very clearly.

**Response:** Vaccinated individuals already have antibodies in their system and including them may bias the results as we may not be able to determine whether they are naturally acquired antibodies or vaccine-induced antibodies.

11. Seropositivity calculated in the Table 3 seems to be wrong. Kindly rectify. For e.g seropositive among who are washing hands should be 1718/2494 which is around 68% not 51.1%? same error is repeated in the entire table

**Response:** This was calculated based on column percentage. Where the denominator is considered as the total number of positives (n=3,476). Hence considering the numerator 1718/3,476, the result is 51.1%.

12. What was the test performance of the kits used? Kindly mention the same in the methods section

**Response**: The WANTAI ELISA Total Antibody kit has a sensitivity of 94.4%, specificity of 100%, a positive predictive value of 100% and a negative predictive value of 95.3%. This has been stated in the methods.

**Reviewer #2**

**Response to main suggestions**

Vaccination was self-reported however this was verified using the vaccination card, thus was not a limitation.

We assume that responses from respondents represent the true state of vaccination status (vaccinated or not). Assuming that an unvaccinated individual for some reason, indicates that he/she was vaccinated when in actual fact he was not vaccinated, the sample size for the analysis will reduce and affect the power of the study. However, this problem was minimized when we adjusted for the non-response rate in the estimation of the sample size.

In the limitations section, it would be helpful to discuss how exclusion of specific “congregate” settings such as prisons, hostels, etc. might affect the population-level estimates, particularly if data or information available about outbreaks in such settings.

**Response**: The study was a nationally representative one. The first stage involves the random sample of enumeration areas and the second stage, random selection of household within the enumeration areas. The study excluded people in prisons, camps, boarding houses, hostels, and hotels, as at the time, government placed restriction in those places. We agree that could be a limitation of the study are we have included that in the limitation section of the discussion.

**Response to minor suggestions**

1. Abstract: suggest adding information about representativeness of the sample

**Response:** This is done.

2. Abstract: indicate the time-period of data collection

**Response**: Time period is indicated.

3. Abstract: would be helpful to indicate the vaccination coverage among the study sample, and clarify that the seroprevalence estimates and risk factors are only among those who were unvaccinated

**Response**: Vaccination coverage in the study sample has been indicated in the text

4. Abstract: the statement “…thirds of the population were exposed to SARS CoV- 2. Seroprevalence was lowest among individuals >60 years” repeats results already presented and could be removed to allow additional word count;

**Response**: Sentence has been removed

5. Abstract: the undiagnosed fraction (or proportion seropositive without a history of a known diagnosed infection) could be helpful to include in results of abstract

**Response**: This has been stated in the abstract

6. Methods: clarify the time-period for measuring adherence (assuming adherence to specific covid protocols are time-variant).

**Response:** The time -period for measuring adherence is the same as the time for samples collection. A questionnaire was administered to each participant which asked questions on adherence.

7. Title: Suggest including “representative sample” in study title. This is a major strength of the study, and highlighting it from the start could be useful.

**Response:** Representative has been included the title

8. Line 41: no need to include the time-stamp as date is sufficient

**Response**: Time stamp has been removed

9. Introduction: suggest adding the vaccine coverage (in addition to the absolute number), line 47

**Response**: Vaccine coverage has been included.

10. Table 4: unclear what the two models refer to (please describe in methods as well as footnote of Table 4).

**Response**: Model 1 is fitted logistic model and Model 2, is a passion model used as sensitivity analysis. We have indicated this in the methods and have effected the suggestion as footnote in Table 4 as suggested by the reviewer.

Please address all correspondence concerning this manuscript to me at [iowusu@noguchi.ug.edu.gh](mailto:iowusu@noguchi.ug.edu.gh).

Thank you for your consideration of this manuscript.

Irene Owusu Donkor, PhD
